# Management of Widespread Pain and Fibromyalgia

**DOI:** 10.1007/s40674-016-0056-5

**Published:** 2016-10-13

**Authors:** Daniel Whibley, Linda E. Dean, Neil Basu

**Affiliations:** Epidemiology Group, Institute of Applied Health Sciences, University of Aberdeen, School of Medicine and Dentistry, Foresterhill, Aberdeen, Scotland AB25 2ZD UK

**Keywords:** Fibromyalgia, Chronic widespread pain

## Abstract

Specialists’ views of fibromyalgia (FM) are typically colored by their experiences of the selected, complex cases that they are regularly called to evaluate. At a population level, it is crucial to recognize that education which promotes patient empowerment and non-pharmacological interventions which support self-management are very effective. The temptation, for both physician and patient, to first reach for pharmacological interventions should be resisted until such holistic approaches are explored. In particular, a strong evidence base supports graded exercise and cognitive behavioral therapies, but such treatments must be intelligently “prescribed.” As reflected by the recent ACR criteria, FM is a highly heterogeneous disorder and is not simply a disorder of pain. For some patients, co-occurring symptoms, such as fatigue, can be equally as impactful and so management strategies should be sufficiently versatile to target those dimensions which are considered priorities at the level of the individual patient. In those patients who do require pharmacological support, patients should not be led to expect significant gains in isolation. The importance of self-management requires emphasis at each and every tier of management. It is true that advances in our understanding of neurobiology have greatly informed the selection of adjunctive drug classes which may provide benefit (as well as those which do not—as is the case of opioids). However, further unpicking of pathogenesis is still required if the FM landscape is to move further towards drug-led management.

## Introduction

Proposed genealogies of the syndrome now known as fibromyalgia (FM) stretch back as far as the late sixteenth century [1]. Key historical moments in its emergence as a distinct entity include the 1904 debut of fibrositis [2] and Hench’s introduction of the term “fibromyalgia” in 1976 [3]. The American College of Rheumatology (ACR) first published classification criteria in 1990 [4••], characterizing FM as chronic widespread pain (pain in the axial skeleton and on both sides of the body, above and below the waist, for at least 3 months), plus 11 out of a possible 18 tender points on clinical examination. The ACR disseminated revised criteria in 2010, the update shifting the emphasis from classification to diagnosis [5••]. The tender point requirement was replaced by recognition of the importance of patient-reported symptoms, including somatic, cognitive, and mood disorders. The revised criteria allowed FM to be measured on a continuous scale that incorporated the widespread-ness of pain and the severity of concurrent symptoms. A modified version of the 2010 criteria was published a year later (2011), and these were used to develop a questionnaire for use in clinical and epidemiological studies of the syndrome [6••]. The mean worldwide prevalence of FM has been estimated to be 2.7 %, with a higher occurrence in women and those of middle or older age and lower socioeconomic status [7]. US prevalence has been estimated as 2.2 % using the 1990 ACR criteria [8] and 6.4 % using the 2010 ACR criteria [9]. Advances in the understanding of the underlying pathophysiology, including recognition of the role of central pain sensitization, have informed FM management. Findings from studies investigating pharmacological, non-pharmacological and complementary, and alternative therapies have been summarized in clinical practice guidelines (CPG). These include The European League Against Rheumatism’s (EULAR) appraisal of the literature, published in 2008 [10•], which is currently being updated. This document was one of six CPGs that contributed to a systematic review of guidelines published up to 2014 [11••] which forms the basis of the evidence provided below.

## Treatment

Given the heterogeneous symptomology of FM, it may be unsurprising that multimodal, multidisciplinary management has been widely advocated. This approach was supported by the highest level of evidence grading (A1: several randomized clinical trials with *p* < 0.01 with meta-analysis) and the strongest possible recommendation (A: benefit significantly outweighs adverse effects and should be applied to all eligible patients) in a recent systematic review of CPGs [11••] that included German and Canadian recommendations [12•, 13•]. Emphasis is placed on patient education and self-management, with the integration of non-pharmacological and then pharmacological treatments as indicated by concomitant, uncontrolled symptoms, including severe pain, mood disorders, sleep problems, and fatigue. Two non-pharmacological treatments have received the highest ratings for level of evidence: exercise and cognitive behavioral therapy (CBT) [11••]. Accordingly, non-pharmacological treatment options are considered first here, followed by pharmacological treatments, and then complementary and alternative therapies. The development and effectiveness of management plans that select from this pool of options would sensibly be stratified according to the individual patient’s priorities. The revised Fibromyalgia Impact Questionnaire (FIQR) is one useful tool which may aid the multi-disciplinary team in the design of bespoke multimodal therapy packages. This reliable and valid multidimensional assessment can also be employed to monitor function, symptoms, and the overall impact of FM over time [14].

### Non-pharmacological treatment

#### Education

Patient education is the keystone of multimodal FM management. The educational component should develop the individual’s understanding of the pathophysiology of FM, especially the recent advances in neurobiology, in the context of a biopsychosocial model of health and illness. Education regarding the importance of self-management that includes information on exercise, sleep habits, and the setting of realistic expectations, should be supported [13•, 15•] and can rarely be achieved during a single session. The effectiveness of patient education has been demonstrated in a number of trials when it is provided as part of a multimodal management package. The provision of an online repository of information and resources which empower patients may be of particular benefit in supporting education, self-management, and improving symptom control and quality of life [11••, 16•].

#### Exercise

Exercise has been identified as effective in managing a range of FM symptoms, including pain, reduced activity, and poor quality of life. A Cochrane review collated the evidence for 47 different types of exercise therapies and determined aerobic exercise to be associated with reductions in pain, number of tender points, and functional limitation [17••]. A later review found similar benefits in response to strengthening exercises [18], and more recent research has supported the effectiveness of aquatic exercise [19]. Given a lack of evidence to support any one specific exercise modality over others, cost, patient preference, and the likelihood of long-term adherence are factors to discuss and agree upon during the development of a management plan. Research that has objectively measured physical activity levels has determined that individuals with chronic pain are less active than those without chronic pain [20]. This relationship may, in part, be explained by beliefs held by individuals with chronic pain regarding movement and symptom exacerbation [21]. Unhelpful beliefs may be given traction if exercise is not introduced in a graded manner, with negative experiences after unpaced exercise deterring future participation. Indeed, the word “exercise” itself may cause trepidation in some individuals with chronic pain, so focusing on increasing activity in general may be a preferable starting point. Guidelines have proposed sessions of moderate to mild intensity activity for a 30-min duration at least two times a week [11••, 12•, 13•]. Garcia et al. found exercise to be included in all CPGs identified up to 2014 and awarded it the highest ratings for both level of evidence and strength of recommendation [11••], highlighting the central role it plays in FM symptom management.

#### Cognitive behavioral therapy

Cognitive behavioral therapy (CBT) can be used to target negative thoughts about pain and its exacerbation on activity and can support behavior change through improvements in symptom management self-efficacy [12•, 13•]. Along with exercise, CBT received the highest rating for level of evidence in a synthesis of CPGs [11••]. A 2013 meta-analysis of 23 randomized controlled trials investigated the impact of CBT on pain, function, and mood in individuals with FM [22••]. Positive, yet modest, effects were observed both at the end of treatment and in the longer term for all outcomes. Evidence suggests that CBT is effective regardless of mode of delivery (one-to-one, group therapy, or internet-based). The choice of medium may be informed by availability, cost-effectiveness, location and, importantly, the patient’s preference and existing commitments (for example, occupational or homecare duties and geographical location) [23]. Consideration of, and response to, these factors may support adherence to a full course of therapy, with consequent associated benefits. An important point to note is that acceptance of CBT may be reduced if it is perceived as a purely psychological intervention [15•]. Instead, CBT as one facet of a multimodal management plan should be fully explained as part of the educational component. It is also too simplistic to consider CBT as a single therapeutic entity which may be “taken off the shelf” and applied uniformly. Existing programs are not necessarily underpinned by the same behavioral models, and perhaps the most optimal strategy is to enable patients to inform the selection of specific themed CBT modules which align best with their individual needs. As an example, some patients may well benefit more from targeting of their fatigue rather than their pain. Such program versatility has been successfully tested and resulted in significant benefits out to 2 years [24]. Finally, CBT programs may be delivered at varying intensities. So called “low grade” CBT has been successfully delivered in many disorders by trained health professionals who do not have a background in psychology [25]. Such an approach is clearly attractive in many health care systems where access to psychologists is limited by costs and/or limited availability.

### Pharmacologic treatment

The pharmacological treatment of FM is largely an “off-label”, testament to the fact that, in the USA, only three drugs are currently Food and Drug Administration (FDA)-approved (pregabalin, duloxetine, and milnacipran). In comparison, two are approved by Health Canada (pregabalin and duloxetine) and none are approved by the European Medicines Agency [26•]. The efficacy of a broad range of pharmacological treatments has been investigated in randomized controlled trials. In general, pharmacological treatment, restricted to the shortest duration possible, should be reserved for cases of severe and uncontrolled symptoms, including pain, sleep, and mood disorders, when non-pharmacological options have been exhausted.

#### Anti-depressants

Classes of anti-depressant frequently used to treat individuals with FM symptoms include tricyclic anti-depressants, Serotonin Norepinephrine Reuptake Inhibitors (SNRIs), and Selective Serotonin Reuptake Inhibitors (SSRIs). Pain reductions resulting from their use are attributed to modulating effects on serotonin and norepinephrine in descending inhibitory pain pathways [27].

##### Tricyclic anti-depressants

Amitriptyline is the most studied anti-depressant in the treatment of FM-related symptoms. Clinical trials have demonstrated reductions in pain, sleep problems, and fatigue, although the methodological quality of studies has been questioned [28]. Improvements have been identified 6 to 8 weeks after commencement at low doses (25 mg/day), but no benefit identified with either an increased dose (50 mg/day) or after a longer duration (12 weeks) when compared to placebo [29]. The Garcia et al. summary of published CPGs rated the level of evidence for amitriptyline as A1, recommending its prescription, at a dose of 10–50 mg/day, for those with severe, uncontrolled pain and sleep problems [11••, 12•, 30•]. Common side effects include drowsiness, dry mouth, and weight gain, and many patients find that such problems outweigh any gains in symptom relief.

##### Serotonin norepinephrine reuptake inhibitors

**Duloxetine**: A systematic review of the efficacy of duloxetine reported a reduction of greater than 30 % for pain effective doses being between 60–120 mg/day, with no difference detected between doses [31•] and lower doses (20–30 mg/day) not demonstrating significant effects. Modest, positive effects on sleep and function have also been reported [32•]. The Spanish and German guidelines, published in 2011 and 2012, respectively [12•, 30•], identified duloxetine as the preferred SNRI, prescribed at a dose of 60 mg/day, for those with comorbid anxiety or depression in cases where amitriptyline is contraindicated or ineffective (level of evidence A1) [11••]. Possible side effects of duloxetine include nausea, palpitations, headache, fatigue, tachycardia, and hypertension [15•].
**Milnacipran**: A Cochrane review conducted by Hauser et al. that investigated the effects of SNRIs on FM symptoms identified five studies that compared milnacipran with placebo [32•]. Its use was associated with a small incremental benefit for pain reduction and slight improvements in fatigue and quality of life but no improvements detected for sleep problems. An effective dose of 100–200 mg/day has been proposed with potential side effects including nausea, palpitations, headache, fatigue, tachycardia, and hypertension [15•]. It has been suggested that, as milnacipran is slightly more noradrenergic than duloxetine, it may be more suitable for individuals presenting with fatigue and memory problems [15•]. However, as it may also be more likely to precipitate hypertension, careful balancing of benefits and risks on a case-by-case basis is required.

##### Selective serotonin reuptake inhibitors

SSRIs used to treat FM symptoms include fluoxetine, paroxetine, and sertraline. In a meta-analysis of the effects of fluoxetine, a moderate effect on pain and modest effects on sleep and fatigue were detected [33]. Side effects of SSRIs include nausea, sexual dysfunction, and weight gain [15•]. Fluoxetine or paroxetine (20–40 mg/day) have been identified by two 2012 guidelines as worthy of consideration for those with comorbid anxiety of depression [12•, 13•].

#### Anticonvulsants

The analgesic effect of anticonvulsants is thought to occur through a reduction in the release of neurotransmitters involved in pain processing, e.g., glutamate and substance P [34]. This class of drugs was developed, and subsequently licensed, as antiepileptics but they are now commonly used in the management of chronic pain [26•].

**Pregabalin**: Pregabalin is FDA-approved for FM symptom management. A Cochrane review from 2013 reported a 30 % reduction in pain but a modest effect on fatigue and sleep and no impact on function [35•]. Standard dosages range from 50–450 mg/day [11••, 12•, 30•], to up to 600 mg/day, in divided doses. Side effects include sedation, weight gain, and dizziness [15•]. Garcia et al. recommend its consideration if amitriptyline is contraindicated or ineffective (level of evidence: A2—several randomized clinical trials have provided evidence *p* < 0.01 without meta-analysis strength of recommendation: B—can be recommended) [11••].
**Gabapentin**: The use of gabapentin for FM symptom management is relatively understudied. A Cochrane review that investigated gabapentin in the treatment of neuropathic pain and FM (date of most recent search January 2011) [36•] included only one study that investigated its effect on FM symptoms [37]. This study reported an improvement in pain (significant 30 % reduction) and function and a modest effect on sleep problems. A dose range of 800–2400 mg/day, in divided doses, has been suggested. Side effects include sedation, weight gain, and dizziness [15•].

#### Nonsteroidal anti-inflammatory drugs (NSAIDs)

There is no evidence to support the efficacy of NSAIDs in managing FM-related pain, with trials consistently demonstrating no benefit over placebo [38]. Indeed, there is evidence to suggest that drugs frequently used to treat peripheral pain, such as NSAIDs and opioids, are not only ineffective, but may exacerbate symptoms [15•]. Given the potentially deleterious side effects affecting the gastrointestinal, renal, and cardiac systems, this class of drugs should only be prescribed in cases of persistent and moderate to severe peripheral pain. In such instances, the lowest effective dose possible should be prescribed for the shortest duration to minimize any secondary effects [15•].

#### Opiods

The apparent hyperactivity of the endogenous opioid system in FM perhaps explains the general inefficacy of opiods [15•]. Tramadol has however been commonly used, although any benefit may actually relate to its mild SNRI effects. Recommendations regarding tramadol use are inconsistent across CPGs. Its use has been associated with significant pain reduction (30 %) [39], although Garcia et al. recommend its use only once all other pharmacological options have been exhausted and in cases of persistent moderate or severe pain [11••]. Tramadol dosage, with or without acetaminophen, has been suggested at 50–100 mg every 6 h. Side effects to be cognizant of include sedation, possible addiction, the development of tolerance, and opioid-induced hyperalgesia [15•].

### Complementary and alternative therapies

In their systematic review of CPGs for the management of FM, Garcia et al. identified four alternative therapies that were recommended by more than one guideline: acupuncture, Qi Gong, Tai Chi, and yoga [11••]. However, given limited corroboration of findings, further research is required to support the effectiveness of these therapeutic options, as well as to determine the benefit of other alternative or complementary approaches. However, as adjuncts to non-pharmacological and pharmacological interventions, patient preference and selection of complementary or alternative therapies may provide a useful way of engaging individuals with active symptom management, encouraging “buy-in” to self-care, and potentially enhancing self-efficacy [15•]. Furthermore, in circumstances where all else fails, patient-directed alternative approaches (as long as they are safe) may most optimally tap into clinically important placebo responses.

## Conclusions

A range of treatment modalities to address complex and heterogeneous FM symptoms should be explored in collaboration with patients. Understanding the individual’s needs and specific symptoms that are restricting activities and participation is key to the development of tailored treatment plans. Education, exercise, and active self-management are highly recommended, providing the core around which pharmacological and non-pharmacological treatments and complementary and alternative therapy adjuncts can be supplemented. Importantly, education should acknowledge the limits of pharmacological treatments in resolving symptoms, and self-management should be promoted within the context of realistic expectation setting. Exercise comes most highly recommended followed by CBT. Short courses of pharmacological treatment are indicated when symptoms are particularly burdensome. Overall, a personalized approach is indicated, where, above the staple of education and encouragement of positive exercise, sleep and stress reduction behaviors, and additional psychological interventions (i.e., CBT) may be of benefit for those with unhelpful coping strategies and pharmacotherapy may be indicated in cases of severe pain, sleep disturbance, or mood disorders. Through actively involving patients in the design of a multimodal management plan that takes into consideration the preferred method and timing of delivery, self-efficacy and a sustained commitment to behavior change may provide the optimal atmosphere for long-term symptom management.
